# Development and validation of a novel immune-related prognostic model in lung squamous cell carcinoma

**DOI:** 10.7150/ijms.47301

**Published:** 2020-06-01

**Authors:** Zeyu Liu, Yuxiang Wan, Yuqin Qiu, Xuewei Qi, Ming Yang, Jinchang Huang, Qiaoli Zhang

**Affiliations:** 1 Third Affiliated Hospital, Beijing University of Chinese Medicine, Beijing, China.; 2 School of Traditional Chinese Medicine, Beijing University of Chinese Medicine, Beijing, China.

**Keywords:** immune-related genes, prognostic model, lung squamous cell carcinoma, The Cancer Genome Atlas, bioinformatics

## Abstract

**Background:** The immune system plays an important role in the development of lung squamous cell carcinoma (LUSC). Therefore, immune-related genes (IRGs) expression may be an important predictor of LUSC prognosis. However, a prognostic model based on IRGs that can systematically assess the prognosis of LUSC patients is still lacking. This study aimed to construct a LUSC immune-related prognostic model by using IRGs.

**Methods:** Gene expression data about LUSC were obtained from The Cancer Genome Atlas (TCGA). Differential expression analysis and univariate Cox regression analysis were performed to identify prognostic differentially expressed IRGs. A prognostic model was constructed using the Lasso and multivariate Cox regression analyses. Then we validated the performance of the prognostic model in training and test cohorts. Furthermore, associations with clinical variables and immune infiltration were also analyzed.

**Results:** 593 differentially expressed IRGs were identified, and 8 of them were related to prognosis. Then a transcription factor regulatory network was established. A prognostic model consisted of 4 immune-related genes was constructed by using Lasso and multivariate Cox regression analyses. The prognostic value of this model was successfully validated in training and test cohorts. Further analysis showed that the prognostic model could be used independently to predict the prognosis of LUSC patients. The relationships between the risk score and immune cell infiltration indicated that the model could reflect the status of the tumor immune microenvironment.

**Conclusions:** We constructed a risk model using four PDIRGs that can accurately predict the prognosis of LUSC patients. The risk score generated by this model can be used as an independent prognostic indicator. Moreover, the model can predict the infiltration of immune cells in patients, which is conducive to the prediction of patient sensitivity to immunotherapy.

## Introduction

Lung cancer is a malignant tumor with a higher global incidence than any other cancer type. In 2018, lung cancer accounted for 11.6% of the total cancer incidence worldwide. It is also the leading cause of cancer-related deaths 1. Lung squamous cell carcinoma (LUSC) is a common pathological type of non-small cell lung cancer (NSCLC), accounting for about 30% of all lung cancers 2. EGFR gene mutations and ALK translocations 3-6 rarely occur in LUSC patients, and thus the options for targeted molecular therapy are limited. Chemotherapy is the primary treatment for patients with advanced LUSC. The median survival time of LUSC patients receiving first-line platinum-containing chemotherapy is only 9-11 months 7, 8.

In recent years, tumor immunotherapy has developed rapidly, especially for immune checkpoint inhibitors that target programmed cell death protein 1 (PD-1) and programmed death-ligand 1 (PD-L1), which have changed the treatment prospects for LUSC patients. PD-1 inhibitors, such as pembrolizumab and nivolumab, improve progression-free survival (PFS) and overall survival (OS) of patients with advanced LUSC 9, 10. However, not all patients benefit from immunotherapy. For example, only about 20% of NSCLC patients respond to anti-PD-1/PD-L1 therapy 11. The expression level of immune-related genes (IRGs) can be used to predict the response to immunotherapy and the patient's prognosis. For example, in patients with squamous cell carcinoma of the head and neck after initial surgery, PDGFRB overexpression is associated with poor prognosis, while patients with PD-1 overexpression have a good prognosis 12. PD-L1 expression is associated with poor prognosis in patients with NSCLC 13, and these patients are more likely to benefit from treatment with immune checkpoint inhibitors 14. However, the molecular biological characteristics of NSCLC of different pathological types are significantly different. Currently, most of the prognostic model studies related to IRGs are aimed at lung adenocarcinoma 15-17. Although there have been some research conclusions regarding IRGs and LUSC prognosis, such as that the cytotoxic lymphocyte antigen 4 (CTLA-4) gene is highly expressed in LUSC patients and patients with a smoking history and predicts poor survival 18, a prognostic model based on IRGs that can systematically assess the prognosis of LUSC patients is still lacking. Therefore, studying the immune-related prognostic markers of LUSC is essential for the implementation of personalized immune precision therapy, prediction of the prognosis, and survival rate improvement for LUSC patients.

In this study, bioinformatics analysis of gene expression data in The Cancer Genome Atlas (TCGA) database was used to screen for differentially expressed IRGs that are intimately related to LUSC, and further detect IRGs significantly associated with the prognosis. By integrating IRGs, a LUSC immune-related prognostic model was constructed that can better evaluate the prognosis of LUSC patients and guide clinical treatment.

## Methods

### Data collection and differential expression analysis

**Figure 1:**
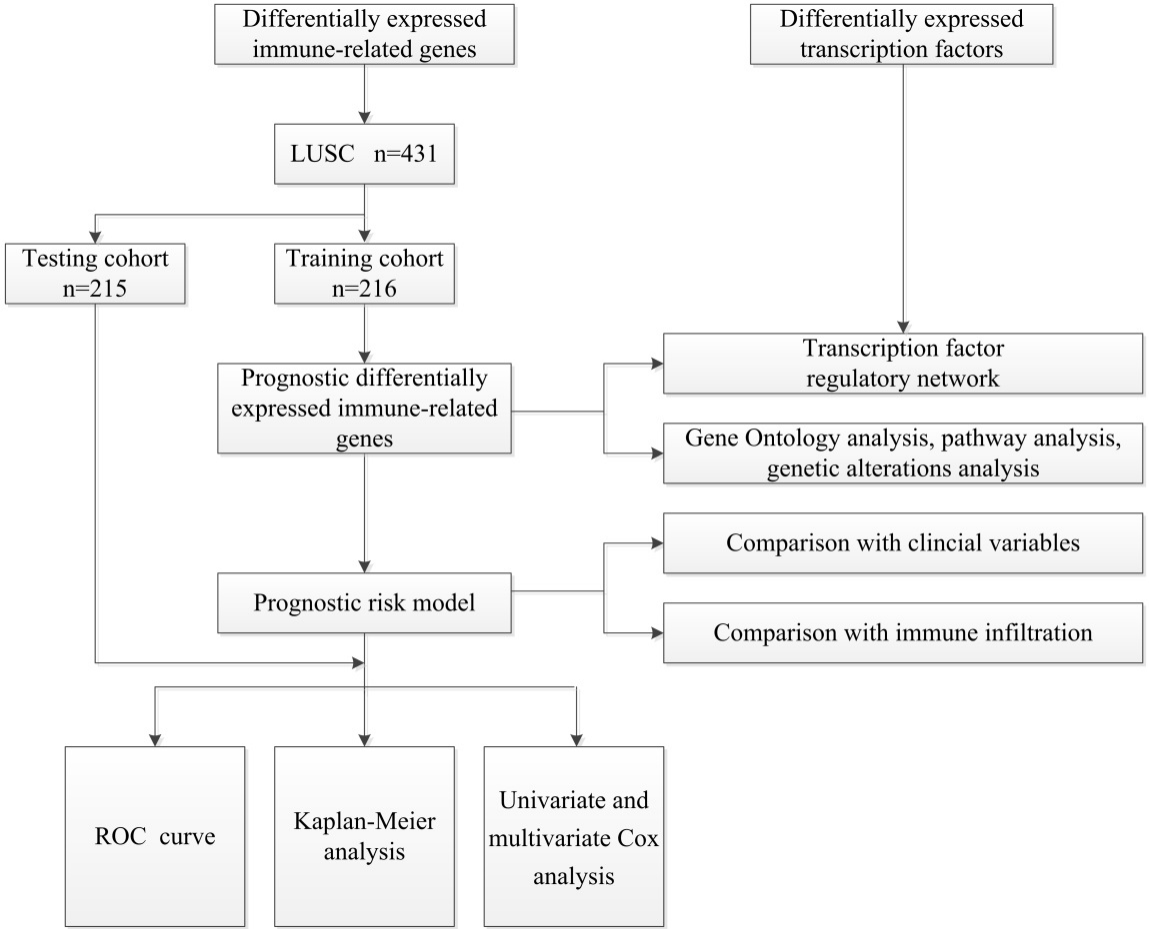
A flow diagram of the overall analysis process.

The overall analysis process is presented in Figure 1. Gene expression data and clinical information about LUSC samples were obtained from the TCGA database (https://portal.gdc.cancer.gov) 19. The RNA-Seq-FPKM data of 502 LUSC patients and 49 non-tumor tissues were downloaded for analysis. Information about immune-related genes was downloaded from the ImmPort database (https://www.ImmPort.org/home) 20. The Cistrome Cancer (http://cistrome.org/CistromeCancer/) is a database for biomedical and genetic research that contains cancer-related transcription factor (TF) data, which we extracted for subsequent study 21. Because the data were downloaded directly from public databases, and we strictly followed the publishing guidelines provided by TCGA, no ethical approval was required.

The limma package of R software 3.6.3 was used for differential expression analysis of the data 22. The Wilcoxon signed-rank test was used to screen for differentially expressed immune-related genes (DEIRGs) and differentially expressed TFs in tumor tissues and normal tissues with cut-off values of FDR < 0.05 and |log2 FC| > 1. Heatmaps were graphed using the pheatmap package.

### Identification and analysis of prognostic DEIRGs

We randomly divided 431 patients with follow-up times of longer than 90 days in the entire TCGA cohort into a training cohort (n = 216) and a test cohort (n = 215). To explore the prognostic value of DEIRGs in LUSC patients, univariate Cox analysis was performed in the training cohort using the survival package. Only genes with *p* < 0.01 were considered as prognostic immune-related genes (PDEIRGs). In order to evaluate the potential biological functions of PDEIRGs, Gene Ontology (GO) 23 enrichment analysis and Kyoto Encyclopedia of Genes and Genomes (KEGG) 24 pathway enrichment analysis were performed using the clusterprofiler package 25 of R software. A *p*-value < 0.05 was set as the screening criterion, and Goplot 26 was used to visualize the results. The cBio Cancer Genomics Portal (cBioPortal, http://www.cbioportal.org/) is an important online platform for analyzing cancer genomics data 27. We used cBioPortal to analyze genetic alterations of PDEIRGs (TCGA, PanCancer Atlas).

### Construction of the transcription factor regulatory network

To evaluate the regulatory effects of TFs on these PDEIRGs, we also studied the correlation between TFs and PDEIRGs. It was performed using the cor.test function in R, whose core method is a Pearson test. Correlation coefficient > 0.5 and *p* < 0.001 were used as cut-off criteria. Cytoscape3.6.0 (http://www.cytoscape.org/) was used to construct the regulatory network and for visualization 28.

### Construction of the prognostic risk model

We used Lasso regression and multivariate Cox regression analysis to evaluate the relationship between PDEIRGs expression and OS, as well as to establish a prognostic model. To calculate the risk score of each patient, the regression coefficients in the multivariate Cox regression model were used to weight the expression values of the selected genes. The risk score is the sum of the expression value of each gene multiplied by the regression coefficient obtained by multivariate Cox regression analysis.

### Validation of the performance of the prognostic model

Patients in the training cohort and test cohort were divided into a high-risk group and a low-risk group according to the median risk score. Kaplan-Meier analysis was performed using the R survival package. The overall survival rates of the high-risk group and the low-risk group were compared by log-rank test, and the receiver operating characteristic (ROC) curve was graphed. An area under the curve (AUC) > 0.60 was considered to be acceptable. Moreover, we used univariate and multivariate analysis to assess whether the risk score generated by our model was independent of other clinical parameters (age, gender, stage, and TNM staging) that are prognostic factors of LUSC.

### Comparison with clinical variables and immune infiltration

To evaluate the model's ability to predict LUSC progression, we analyzed the relationship between risk factors (risk scores and risk genes) in the model and clinical variables (age, gender, stage, and TNM staging). Tumor Immune Estimation Resource (TIMER, http://cistrome.dfci.harvard.edu/TIMER/) is a database for comprehensive analysis of tumor-infiltrating immune cells 29. We used it to study the correlation between the prognostic model's risk score and tumor-infiltrating immune cells.

## Results

### Data collection and differential expression analysis

**Figure 2:**
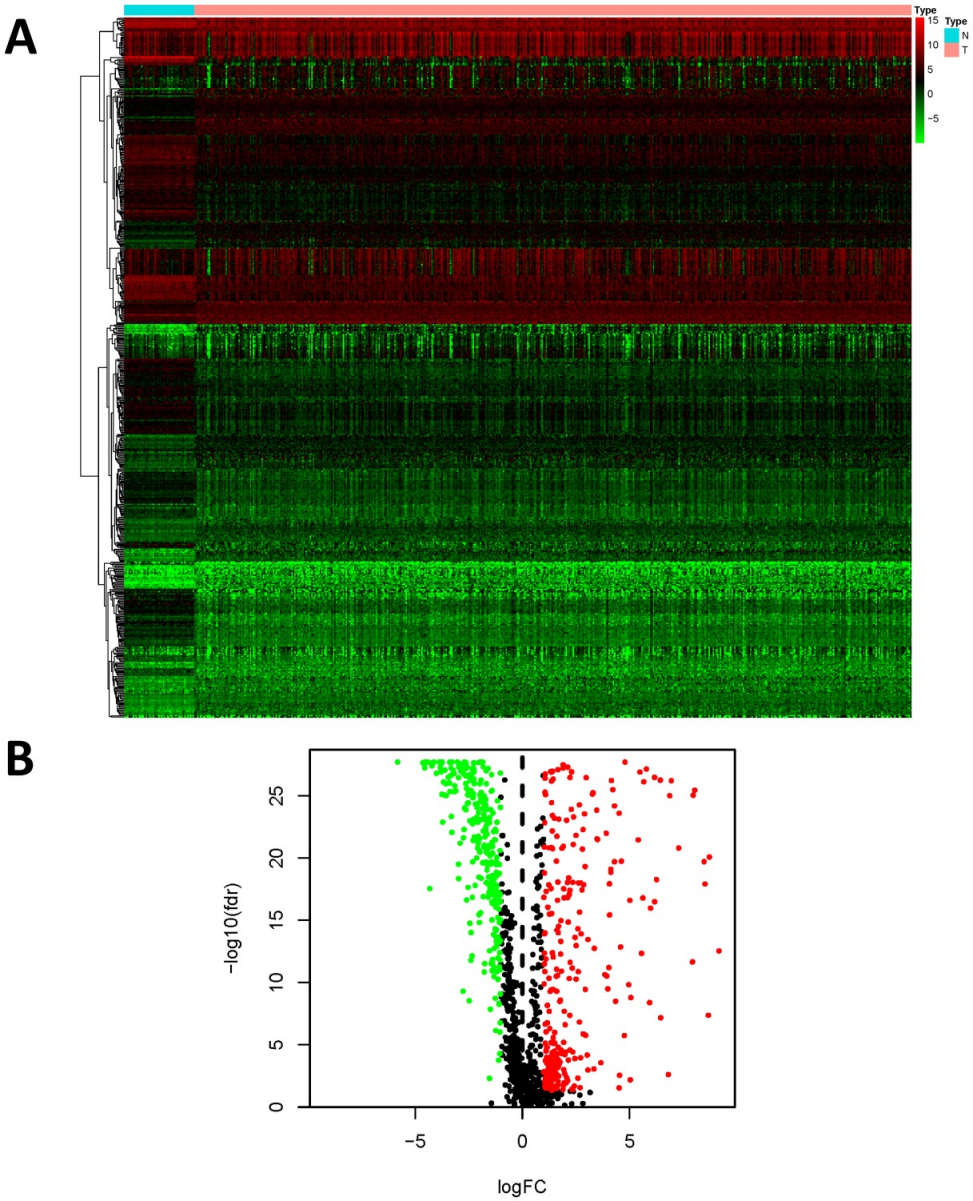
Differentially expressed immune-related genes (DEIRGs). (A) Heatmap of DEIRGs; the green to red spectrum indicates low to high gene expression. (B)Volcano plot of DEIRGs; the green dots represent downregulated genes, the red dots represent upregulated genes and the black dots represent genes that were not significantly differentially expressed.

We examined the gene expression level of 2498 IRGs in LUSC tissues (n = 502) and non-tumor tissues (n = 49) in TCGA, and identified 593 DEIRGs (Figure 2), among which 307 genes were upregulated, and 286 genes were downregulated in LUSC tissues (FDR < 0.05 and |log2FC| > 1).

### Identification and analysis of prognostic DEIRGs

The 431 patients with a follow-up time of longer than 90 days in the entire TCGA cohort were randomly divided into the training cohort (n = 216) and the test cohort (n = 215). To determine PDEIRGs, we performed univariate Cox regression analysis on the expression of each indicator in the training cohort. A total of 8 DEIRGs were identified that were significantly associated with OS in LUSC patients (*p* < 0.01) (Table 1).

**Figure 3:**
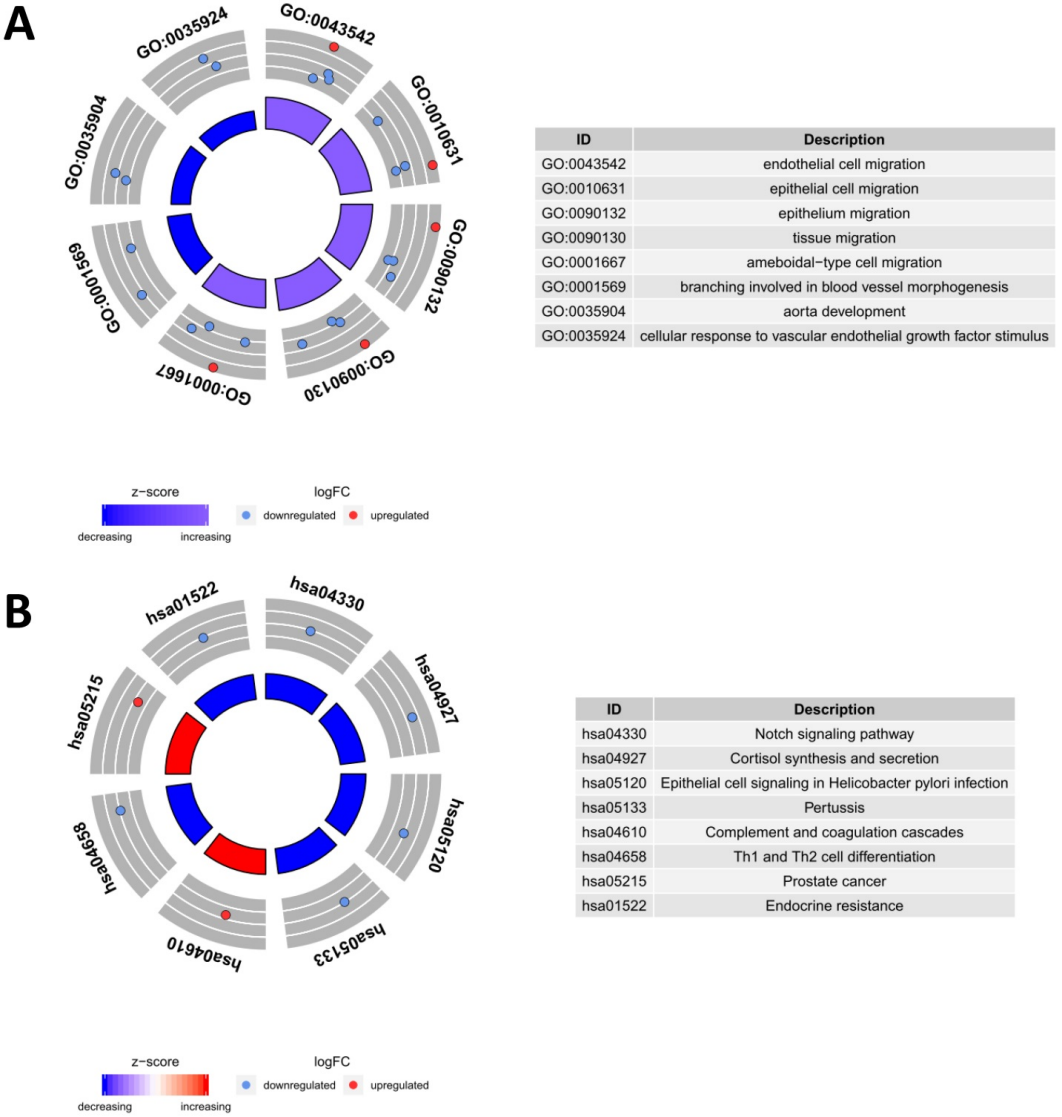
Functional enrichment analysis of prognostic differentially expressed immune-related genes (PDEIRGs) in LUSC. The outer circle represents the expression (logFC) of PDEIRGs in each enriched GO (gene ontology) term (A) or pathway (B): red dots indicate upregulated PDEIRGs and blue dots indicate downregulated PDEIRGs. The inner circle indicates the significance of GO terms (A) or pathways (B) (log10-adjusted P values).

**Figure 4:**
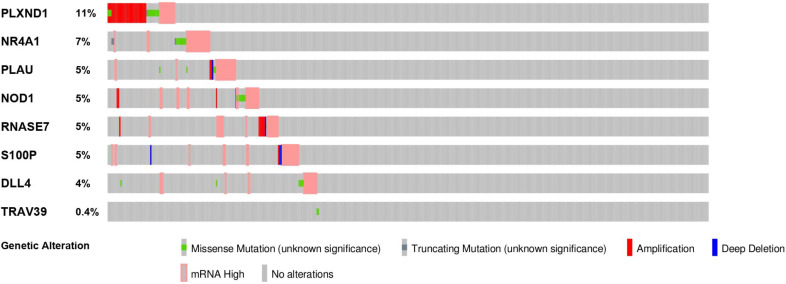
The genetic alteration of 8 genes in LUSC patients using the cBioPortal database.

To evaluate the potential biological functions of the PDEIRGs, we performed GO enrichment analysis, KEGG pathway enrichment analysis, and genetic alteration analysis. The GO enrichment analysis results showed that PDEIRGs were enriched in multiple BP (biological process), MF (molecular function), and CC (cellular component) terms (Table 2, Figure 3A). When sorted according to the *p* values, the top BP terms are endothelial cell migration and epithelial cell migration. The top MF terms are peptidoglycan binding, glycosaminoglycan binding, and RAGE receptor binding, and the top CC terms are semaphorin receptor complex, cell projection membrane, and lamellipodium membrane. Pathway enrichment analysis showed that genes were primarily intimately related to the Notch signaling pathway, cortisol synthesis, and secretion, and epithelial cell signaling in Helicobacter pylori infection (Table 3, Figure 3B). The cBioPortal tool was used to analyze the genetic alterations of 8 PDEIRGs. As shown in Figure 4, PLXND1 and NR4A1 are the most commonly altered genes. Of the 466 samples, 164 (35%) samples demonstrated genetic alterations, whose changes were mainly “mRNA high” and “amplification”. Eight immune-related prognostic genes (NOD1, PLAU, TRAV39, RNASE7, S100P, NR4A1, DLL4, PLXND1) were altered.

### Construction of the transcription factor regulatory network

**Figure 5:**
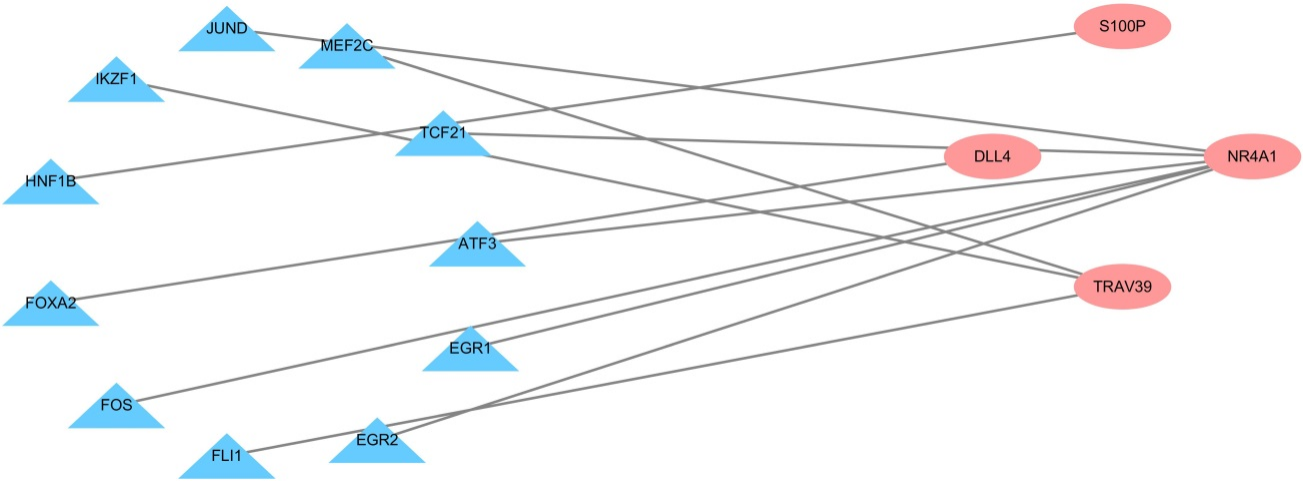
Transcription factor regulatory network. The pink nodes represent PDEIRGs and the blue nodes represent transcription factors that correlated with the PDEIRGs in terms of their mRNA levels (correlation coefficient > 0.5 and *p* < 0.001).

To explore the possible mechanism of dysregulated PDEIRG expression in LUSC, we analyzed the correlation between TFs and PDEIRG expression. First, we examined the expression levels of TFs in LUSC tissues (n = 502) and normal tissues (n = 49), and identified 111 TFs (FDR < 0.05 and |log2 FC| > 1) that were significantly differentially expressed between the two tissue types. Subsequently, we used correlation coefficient > 0.5 and *p-*value < 0.001 as cut-off values to analyze the correlation between the 111 TFs and the mRNA levels of PDEIRGs. Eleven TFs were significantly associated with the abnormal expression of four PDEIRGs. To better interpret the regulatory relationship, we constructed a regulatory network based on TFs. As shown in Figure 5, there were eleven transcription factors (JUND, MEF2C, TCF21, ATF3, EGR1, EGR2, FLI1, FOS, FOXA2, HNF1B, IKZF1) and four PDEIRGs (S100P, NR4A1, TRAV39, DLL4) in the network.

### Construction of the prognostic risk model

**Figure 6:**
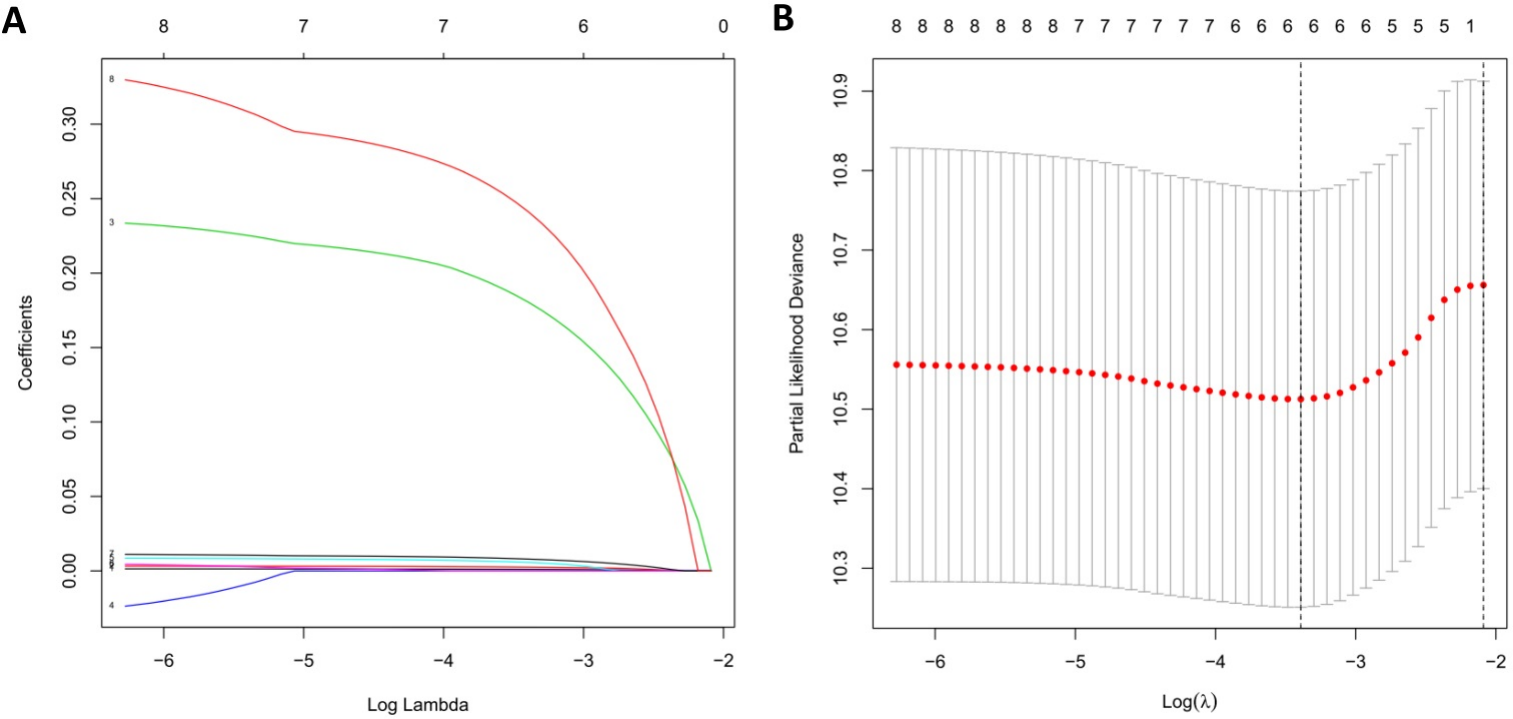
LASSO regression. (A) The changing trajectory of each independent variable. The horizontal axis represents the log value of the independent variable lambda, and the vertical axis represents the coefficient of the independent variable. (B) Confidence intervals for each lambda.

Due to the impact of PDEIRGs on the patient's OS, PDEIRGs were further screened to construct a Cox regression risk model. First, to avoid overfitting the model, we used Lasso regression to remove PDEIRGs that were highly correlated with each other (Figure 6). We obtained six candidate PDEIRGs, and further analyzed them through multivariate Cox proportional hazards regression analysis (with forward selection and backward selection). Finally, we obtained four optimal PDEIRGs to be included in the risk prediction model: S100P, PLAU, NOD1, and TRAV39 (Table 4). These genes were confirmed to be high-risk genes of OS for the patients, predicting poor prognosis.

In order to explore the significance of these risk genes in predicting the prognosis of LUSC patients, we used the expression level of these risk genes and regression coefficients to calculate the risk score of each patient. The calculation formula is as follows: risk score = (0.0015 × S100P expression) + (0.0038 × PLAU expression) + (0.2667 × NOD1 expression) + (0.3211 × TRAV39 expression).

### Validation of the performance of the prognostic model

**Figure 7:**
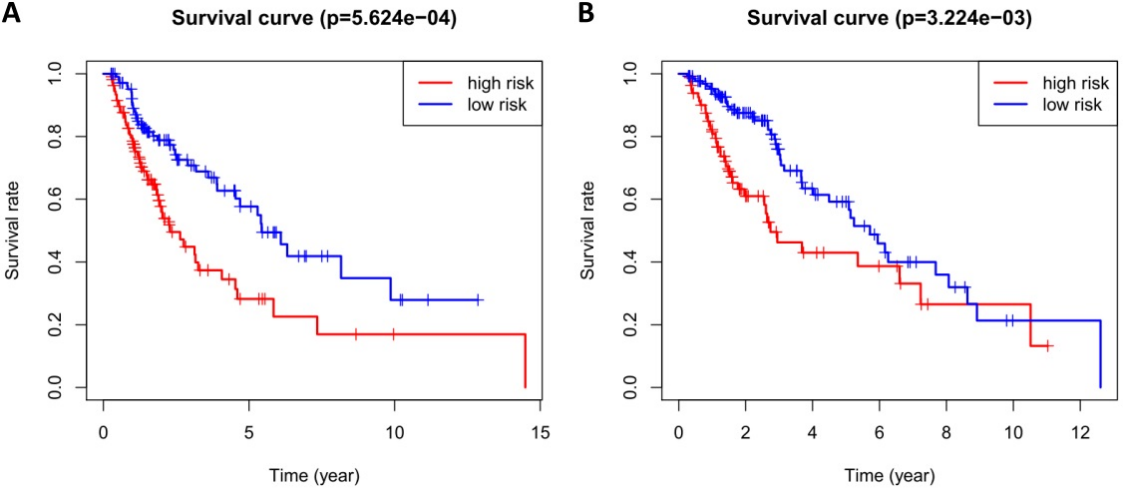
Kaplan-Meier curve analysis for overall survival of training cohort (A) and test cohort (B) using the 4 genes signatures.

**Figure 8:**
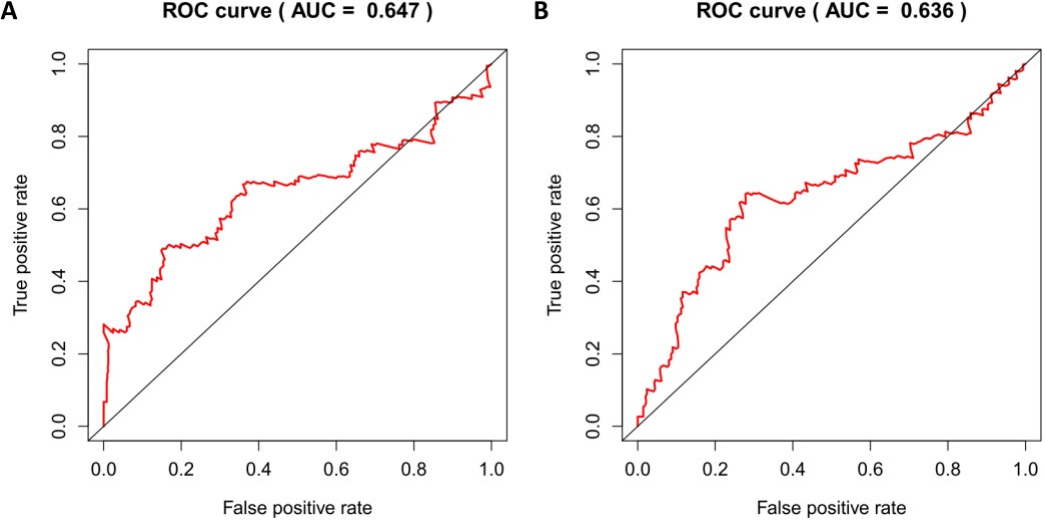
Time-dependent ROC curves analysis for 3-year survival prediction of training cohort (A) and test cohort (B) by the prognostic model.

**Figure 9:**
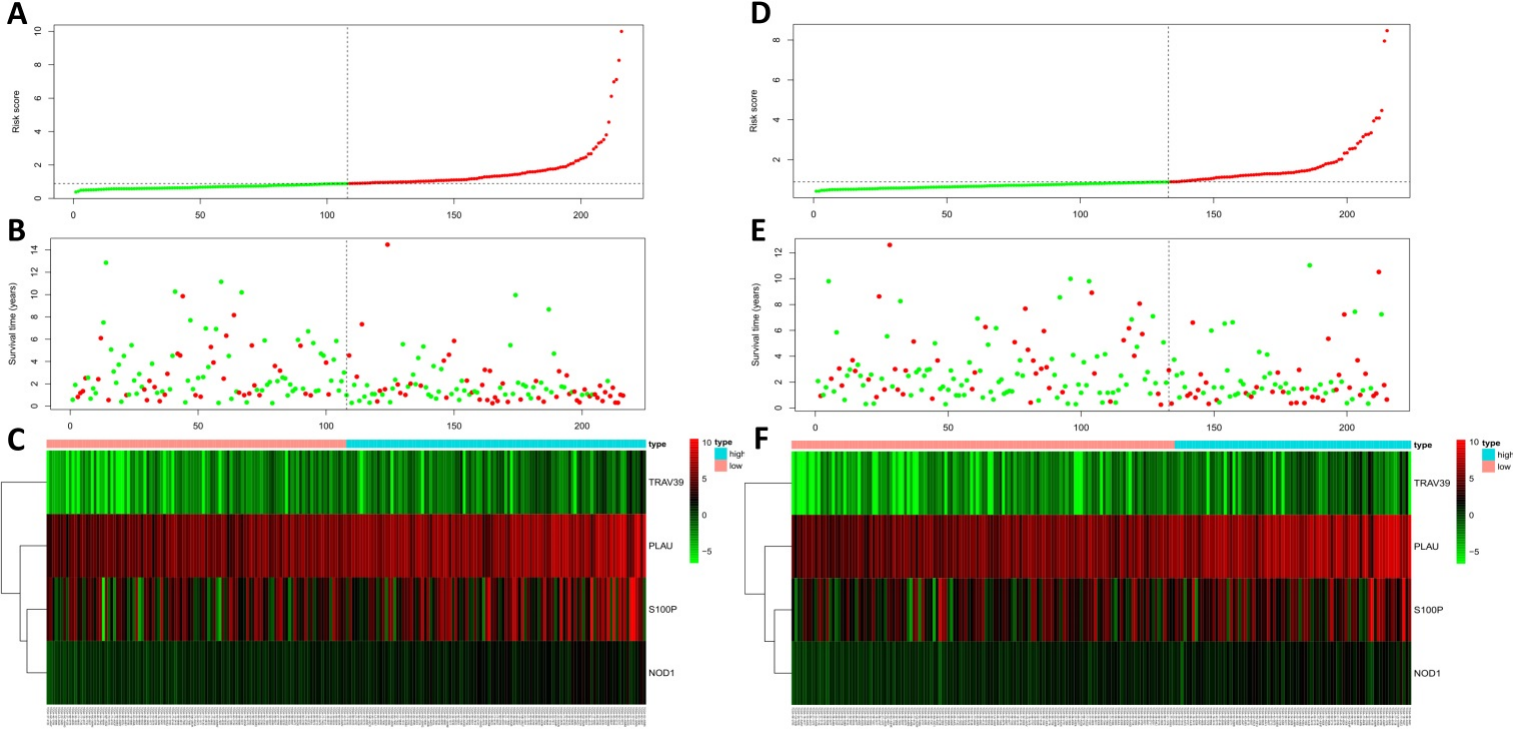
Risk score analysis of training cohort (A, B, C) and test cohort (D, E, F). (A, D) Rank of risk score and distribution of groups. (B, E) The survival status of LUSC patients in different groups. (C, F) Heatmap of the 4 key immune-related genes. The color from green to red shows an increasing trend from low levels to high levels.

According to the median risk score, patients in the training cohort were divided into a high-risk group and a low-risk group. To determine the prognostic difference between the high-risk group and the low-risk group, we established Kaplan-Meier curves based on a log-rank test. The prognosis of the high-risk group was worse than that of the low-risk group (*p* < 0.05) (Figure 7A). We then used a time-dependent receiver operating characteristic (ROC) curve to assess the prediction accuracy of the model. The three-year AUC value of the prediction model was 0.647 (Figure 8A). Subsequently, we sorted the risk scores of patients in the training cohort and analyzed their distribution. The survival status of each patient is labeled in Figure 9A. A heatmap was graphed to describe the expression pattern of the risk genes in the high-risk and low-risk groups (Figure 9C). In patients with high-risk scores, four risk genes (S100P, PLAU, NOD1, and TRAV39) were upregulated (Figure 9A). These risk genes showed an opposite expression pattern in patients with low-risk scores.

To verify the accuracy of the prediction model, we used it to analyze the test cohort. First, we used the four risk genes (S100P, PLAU, NOD1, and TRAV39) to calculate the risk score for each patient in the test cohort. These patients were then divided into two groups according to how their risk score compared to the median risk score of the training cohort. Kaplan-Meier survival analysis was used to determine the prognostic difference between the high-risk and low-risk groups. In the test cohort, the Kaplan-Meier survival curves of the two risk groups were significantly different (*p* < 0.05) (Figure 7B), and the three-year AUC was 0.636 (Figure 8B). The distribution of risk scores, survival status, and risk gene expression of the test cohort are shown in Figure 9D, E, F. Similar to the results of the training cohort, the risk gene expression levels in the low-risk group were lower than those in the high-risk group. These results indicate that our prognostic risk model can accurately predict the prognosis of LUSC patients.

**Figure 10:**
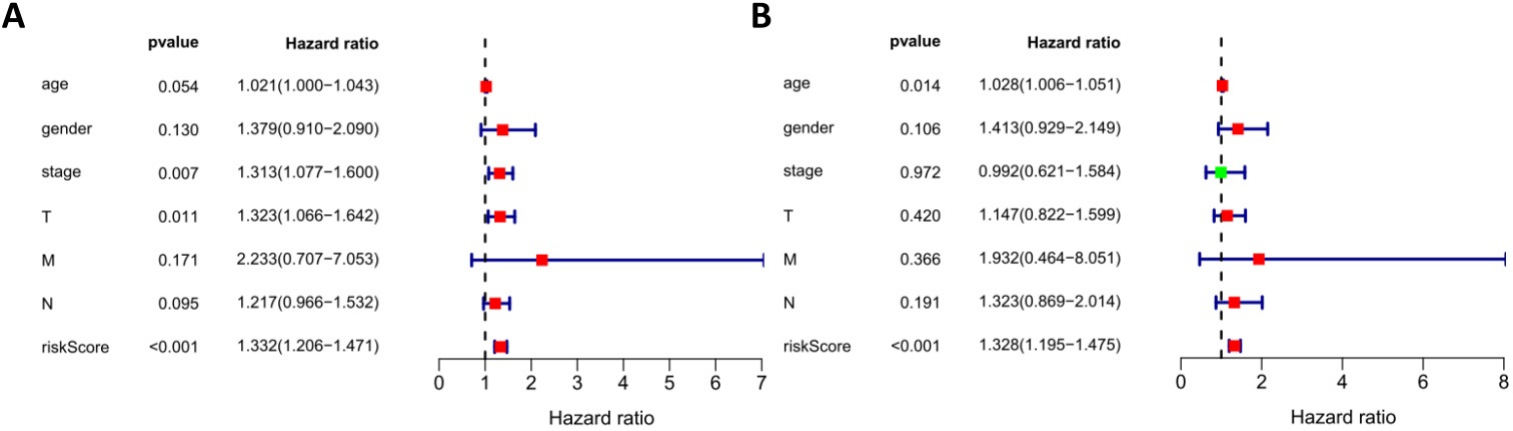
Univariate and multivariate analyses of overall survival in LUSC patients of TCGA. (A) Univariate analysis. (B) Multivariate analysis.

Next, we performed univariate and multivariate Cox regression analysis to assess whether the risk score generated by our model was independent of other clinical parameters (age, gender, stage, and TNM staging) that are prognostic factors for LUSC. Univariate analysis showed that stage, T staging, and risk score were correlated with the prognosis of LUSC patients (Figure 10A). Multivariate analysis showed that the risk score was independently correlated with OS in the entire TCGA cohort (*p* < 0.05) (Figure 10B). These results suggest that the prognostic risk model can be used independently to predict the prognosis of LUSC patients.

### Associations with clinical variables and immune infiltration

**Figure 11:**
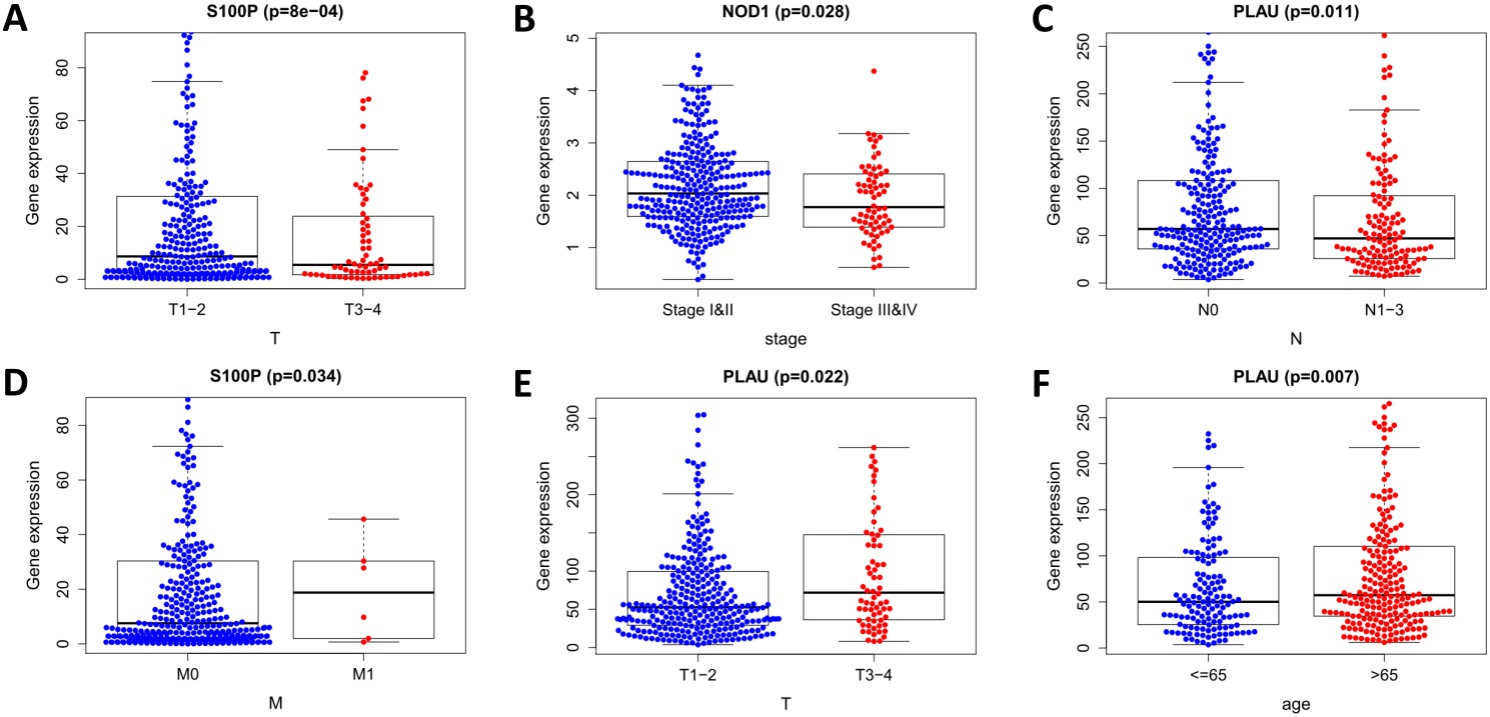
Relationships of the variables in the model with the clinical characteristics of patients in the entire TCGA cohort. (A) S100P expression and T stage. (B) NOD1 expression and pathological stage. (C) PLAU expression and N stage. (D) S100P expression and M stage. (E) PLAU expression and T stage. (F) PLAU expression and age.

We analyzed the relationship in the model between risk factors (risk scores and risk genes) and clinical variables (age, gender, stage, and TNM staging) (Figure 11). With the increase of PLAU expression level, the number of LUSC patients in the T stage increased, while the number of LUSC patients in the N stage decreased (both *p* < 0.05). As the S100P expression level increased, the number of LUSC patients in the M stage increased, while the number of LUSC patients in the T stage decreased (both *p* < 0.05). With the increase of NOD1 expression level, the staging of LUSC patients decreased (*p* < 0.05). The expression of PLAU was higher in patients over the age of 65 than it was in patients under 65 (*p* < 0.05). These results indicate that the dysregulated expression of immune-related risk genes is related to the occurrence and development of LUSC.

**Figure 12:**
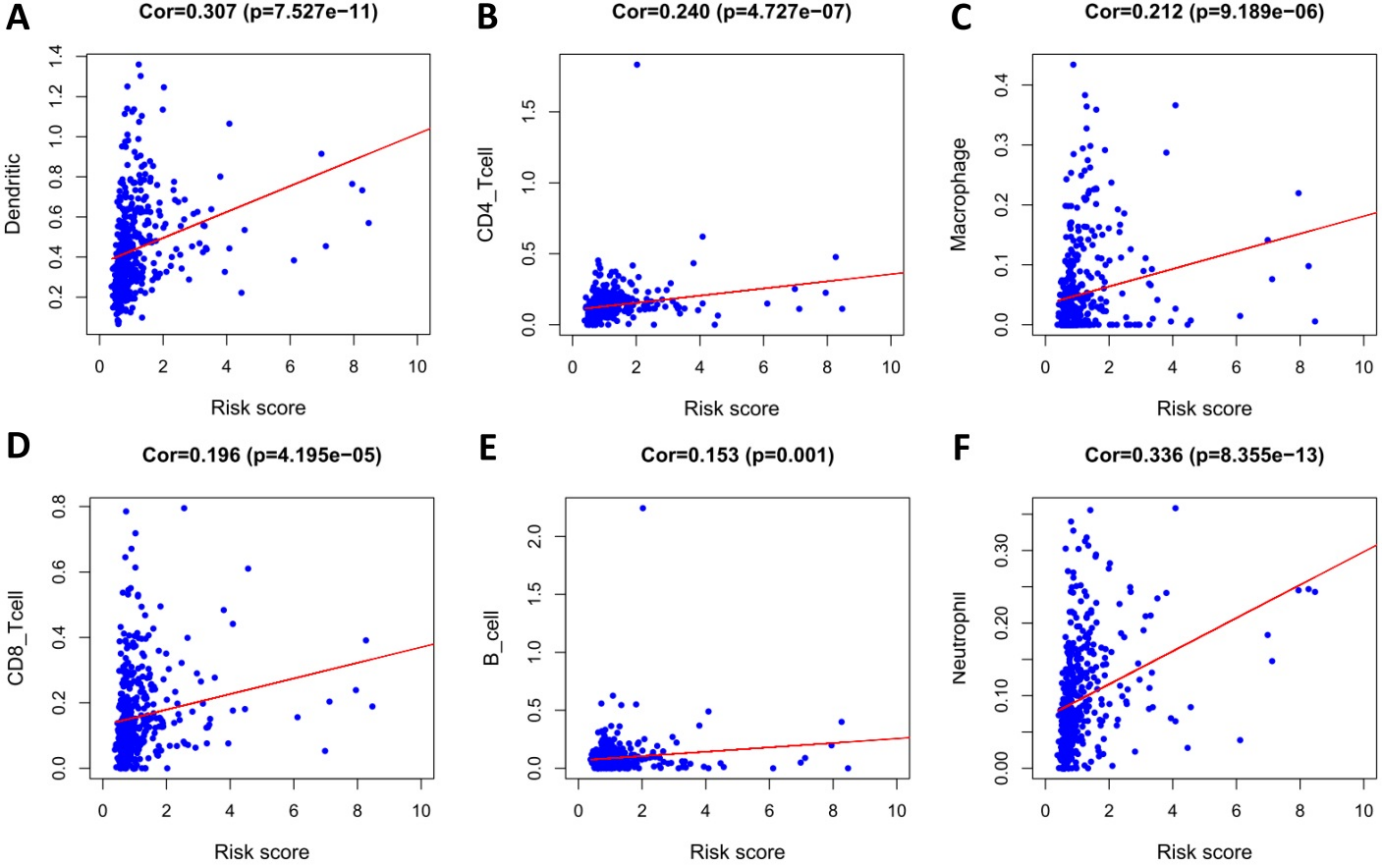
Analysis of the correlation between the risk score and immune cell infiltration in the entire TCGA cohort. (A) Dendritic cells. (B) CD4+ T cells. (C) Macrophages. (D) CD8+ T cells. (E) B cells. (F) Neutrophils.

To determine whether our model can reflect the status of the patient's tumor immune microenvironment, we analyzed the correlation between risk scores and immune cell infiltration. With the increase of risk score, the content of six types of immune cells (B cells, CD4+ T cells, CD8+ T cells, neutrophils, macrophages, and dendritic cells) in LUSC tissues increased (*p* < 0.05) (Figure 12).

## Discussion

The immune system plays an important role in the occurrence and development of cancer. Therefore, IRG expression may be an essential predictor of LUSC progression and prognosis. The importance of the IRG model in predicting the prognosis of cancer patients has been described in previous studies 30. In this study, we identified IRGs related to prognosis and constructed a reliable model using them to predict the prognosis of LUSC patients.

This study analyzed the gene expression data of LUSC patients in TCGA, identified 593 DEIRGs, and then found eight DEIRGs whose expression was related to OS using univariate Cox regression analysis. These results indicate that IRGs are an important prognostic factor for LUSC patients. The results of GO enrichment analysis showed that immune-related prognostic genes were mainly related to the proliferation of endothelial cells and epithelial cells. Pathway analysis results showed that the genes were focused on several pathways related to cancer and immunity.

In order to explore the molecular mechanism of the abnormal PDEIRG expression, we constructed a TF regulatory network and found that 11 TFs were related to the expression of PDEIRGs. These results indicate that TFs determine the impact of PDEIRGs on a patient's OS. Moreover, some studies have confirmed that these TFs are closely related to the occurrence and development of tumors. For example, recent studies have found that FLI1 can regulate the transcriptional activity of target genes through binding to the promoters or enhancers of multiple genes via specific sequences to play a cancer-promoting effect 31, 32. A growing number of studies have shown that FLI1 is abnormally highly expressed in a variety of solid tumors and is intimately associated with both tumorigenesis and tumor development 33, 34. TCF21 is one of the important members of the basic helix-loop-helix (bHLH) family. Proteins of this family often form dimers and bind to DNA promoter regions, thereby regulating the expression of downstream genes 35. Studies have shown that the TCF21 gene regulates the differentiation of mesenchymal cells to epithelial cells, that is, the reversal of the epithelial-mesenchymal transition process. This function is lacking in tumor tissues and has important significance to cell growth and differentiation 36. Richards *et al.* showed that high methylation and low expression of TCF21 were very common in NSCLC, and were detected even in the early stage of the disease, which makes TCF21 one of the potential markers for early NSCLC screening 37. The TF regulatory network will provide the basis for future research into the molecular mechanism of LUSC.

Subsequently, through Lasso regression and Cox regression analysis, we identified four PDEIRGs of interest (S100P, PLAU, NOD1, and TRAV39) and used them to construct a prognostic model. Previous studies have shown that S100P is abnormally expressed in many tumors, and its expression level is related to the staging and prognosis of some tumors 38-40. Diederichs *et al.* have confirmed that S100P expression is associated with metastasis and predicts survival in early stages of NSCLC 41. Our study also found that S100P was positively correlated with risk scores of LUSC patients. Ning *et al.* found that PLAU was upregulated in the early, middle, and advanced stages of LUSC compared with paracancer tissues 42. Moreover, the upregulation of PLAU is associated with increased adverse outcomes in patients, suggesting that PLAU may be a new biological marker or potential therapeutic target for LUSC, which is consistent with our findings. Studies on the relationship between NOD1 and NSCLC are still rare. Some studies claimed that genetic variations in the NOD1 gene have been found to be associated with lung cancer risk 43. However, studies of the relationship between NOD1 and LUSC prognosis are still lacking. Currently, no related studies have reported the effect of TRAV39 on LUSC. The role of these potential IRGs in LUSC awaits further study.

Furthermore, we also analyzed the reliability and stability of the model. The results showed that the model could accurately distinguish patients with different survival outcomes. Univariate and multivariate Cox regression analysis showed that the model could independently predict the prognosis of patients. Therefore, our model can be used to identify LUSC patients at a high risk of death, enabling early intervention in clinical practice to improve a patient's prognosis.

We also analyzed the relationship between factors in the model and certain clinical variables (age, gender, pathological stage, and TNM staging), and found that multiple factors in the model (such as the expression of PLAU, S100P, and NOD1) were related to the progression of LUSC. Therefore, the risk model is of high clinical application value for the prediction of LUSC progression.

Previous studies have shown that immune infiltration is closely associated with tumor response to treatment and prognosis. Therefore, in order to explore the status of tumor immune microenvironment, we analyzed the relationship between risk score and immune cell infiltration, and found that the risk score was positively associated with the infiltration of six types of immune cells (B cells, CD4+ T cells, CD8+ T cells, neutrophils, macrophages, and dendritic cells). Regarding T cells, it is currently believed that high CD4+ T lymphocyte infiltration in tumor stroma, rather than in tumor cell nests, is correlated with better OS in lung cancer patients 44, while regulatory T cells are associated with a poorer prognosis 45. However, there is no consistent conclusion about the relationship between the infiltration of CD8 T cells in tumor tissues and prognosis 46, 47. Data show that among stage III NSCLC patients receiving chemoradiotherapy, patients with higher CD8+ tumor-infiltrating lymphocyte density in pre-treatment biopsy specimens had longer PFS and OS 48. This study suggests that the infiltration of CD8+ T and CD4+ T cells is positively correlated with the risk score, which may be related to different T cell subpopulations. The specific reasons for this await further study.

Studies have shown that tumor-associated B cells and activated STAT3 can promote tumor progression through regulating angiogenesis 49. The relationship between B cell infiltration in NSCLC and a patient's prognosis is not clear 45, and some studies on NSCLC failed to detect an effect of B cell density on prognosis 50, 51. In fact, B cells in NSCLC may play different roles, such as antibody specificity, antigen presentation, and immunosuppression 52. Therefore, different B cell subpopulations may have different effects on tumors. The specific effects require more detailed research. Tumor-associated neutrophils have been shown to be associated with poor prognosis in a variety of cancers 53. For NSCLC, studies have demonstrated that increased density of tumor-associated CD66b+ neutrophils is correlated with adverse prognostic factors, but not directly correlated with patient outcomes 54. This is consistent with our findings. Chen *et al.* reported that tumor-associated macrophages (TAMs) were associated with a poor prognosis in NSCLC 55. Welsh *et al.* found that TAM infiltration in the cancer islet was correlated with a good prognosis, while TAM infiltration in the matrix was associated with a poor prognosis 56. Furthermore, studies have shown that mature dendritic cell number in tumor infiltration is positively related to the survival time of patients, while our results found that dendritic cell infiltration and risk score were positively correlated 57. This discrepancy may be due to the fact that mature dendritic cells are not a major component of tumor-infiltrating dendritic cells. These results suggest that this model can be used as a predictor of immune cell infiltration.

In this study, we studied the expression pattern of IRGs in LUSC. Secondly, we used multiple algorithms (including univariate Cox, multivariate Cox, and Lasso regression) to identify PDEIRGs, and used a test cohort to verify the reliability of the risk model. Our analysis demonstrated that our results are reliable. Inevitably, our study has certain limitations. We used data from public databases that have not been validated in prospective clinical studies. Moreover, the identified mechanisms by which IRGs affect LUSC require verification by *in vivo* and *in vitro* studies.

## Conclusion

We constructed a risk model using four PDIRGs that can accurately predict the prognosis of LUSC patients. The risk score generated by this model can be used as an independent prognostic indicator to distinguish patients with different survival outcomes. Moreover, the model can predict the infiltration of immune cells in patients, which is conducive to the prediction of patient sensitivity to immunotherapy. However, further experiments are needed to validate the results of this study.

## Figures and Tables

**Table 1:** Prognostic differentially expressed immune-related genes in LUSC.

ID	HR	Low 95%CI	High 95%CI	*p* value
NOD1	1.347935	1.124713	1.61546	0.001228
PLAU	1.003822	1.001486	1.006164	0.001333
TRAV39	1.630708	1.183324	2.247235	0.002801
RNASE7	1.013658	1.004548	1.022851	0.003228
S100P	1.001283	1.000387	1.002179	0.004988
NR4A1	1.015771	1.004139	1.027537	0.007746
DLL4	1.137407	1.034404	1.250668	0.007852
PLXND1	1.041016	1.010496	1.072457	0.008104

**Table 2:** Functional enrichment analysis of prognostic differentially expressed immune-related genes in LUSC.

Ontology	ID	Description	*p* value
BP	GO:0043542	endothelial cell migration	2.99E-06
BP	GO:0010631	epithelial cell migration	8.10E-06
BP	GO:0090132	epithelium migration	8.38E-06
BP	GO:0090130	tissue migration	8.95E-06
BP	GO:0001667	ameboidal-type cell migration	2.37E-05
CC	GO:0002116	semaphorin receptor complex	0.004455
CC	GO:0031253	cell projection membrane	0.007972
CC	GO:0031258	lamellipodium membrane	0.008893
CC	GO:0031528	microvillus membrane	0.009296
CC	GO:0070821	tertiary granule membrane	0.029243
MF	GO:0042834	peptidoglycan binding	1.82E-05
MF	GO:0005539	glycosaminoglycan binding	0.003354
MF	GO:0050786	RAGE receptor binding	0.004344
MF	GO:0017154	semaphorin receptor activity	0.004738
MF	GO:0008656	cysteine-type endopeptidase activator activity involved in apoptotic process	0.006313

If there were more than five terms in this category, selected the first five terms based on the *p* value.

**Table 3:** Pathway analysis of prognostic differentially expressed immune-related genes in LUSC.

ID	Description	*p* value
hsa04330	Notch signaling pathway	0.026181
hsa04927	Cortisol synthesis and secretion	0.032037
hsa05120	Epithelial cell signaling in Helicobacter pylori infection	0.034469
hsa05133	Pertussis	0.037381
hsa04610	Complement and coagulation cascades	0.041738
hsa04658	Th1 and Th2 cell differentiation	0.045116
hsa05215	Prostate cancer	0.047523
hsa01522	Endocrine resistance	0.048004
hsa04925	Aldosterone synthesis and secretion	0.048004

**Table 4:** Multivariate Cox regression analysis of 4 genes associated with overall survival in LUSC patients.

ID	Coef	HR	Low 95%CI	High 95%CI	*p* value
S100P	0.001498	1.0015	1.000563	1.002437	0.0017
PLAU	0.003828	1.003836	1.001598	1.006078	0.000771
NOD1	0.266728	1.305686	1.066776	1.5981	0.009685
TRAV39	0.321082	1.378618	0.965213	1.969087	0.077513
